# Noise exposure assessment of non-coal mining workers in four provinces of China

**DOI:** 10.3389/fpubh.2022.1055618

**Published:** 2023-01-09

**Authors:** Xin Wang, Ning Kang, Yiwen Dong, Kai Liu, Kang Ning, Hongying Bian, Feng Han, Yongqing Chen, Meng Ye

**Affiliations:** ^1^National Institute of Occupational Health and Poison Control, Chinese Center for Disease Control and Prevention, Beijing, China; ^2^National Center for Occupational Safety and Health, Beijing, China; ^3^Liaoning Provincial Center for Disease Control and Prevention, Shenyang, China

**Keywords:** non-coal mine, individual noise, position, exposure, HPD

## Abstract

**Objective:**

This study aimed to understand the noise exposure of non-coal mines in China to take appropriate controls to protect workers' health.

**Methods:**

An assessment of non-coal miners' noise exposures was conducted in four provinces in China. Individual noise exposure levels were measured, and the survey on the hearing protector device (HPD) equipment was administered.

**Results:**

423 noise dosimeter measurements were obtained, including drilling, blasting, ore drawing, transportation, winching, crushing, screening and ball milling, and auxiliary (air pressure, pump, and maintenance). A total of 31.9% of the individual noise levels (L_EX,8h_) exceeded 85 dB(A), and the median dosages of non-coal miners with high noise exposure were: excavation workers-89.1 dB(A), mill operators-88.7 dB(A), and crusher operators-87.0 dB(A). The noise dose of underground mine workers is higher than that of surface mine workers (*P* < 0.001). A total of 53.7% of non-coal mining enterprises are not equipped with HPD for workers, mainly small and micro enterprises.

**Conclusions:**

High levels of hazardous noise exposure are typical in non-coal mines. Noise exposure data can help to develop more feasible noise controls.

## Introduction

Noise is one of the most common occupational hazards, and overexposure to noise continues to be a problem throughout the mining industry (1). Occupational hearing loss is a common work-related illness among mining workers: miners work in a high-noise environment for a long time, and their hearing gradually decreases (2–4). It takes several hours or even longer to recover their hearing after leaving the environment. If they continue to work in such an environment without noise controls, it will cause permanent hearing threshold displacement, resulting in irreversible hearing loss and even noise-induced deafness. In general, noise levels above 85 dB(A) are considered hazardous, depending on the time and frequency of noise exposure and hearing protector device (HPD) use.

In recent years, the degree of mining mechanization has increased, and many large, efficient, and high-power pieces of equipment have been widely used. While improving production efficiency, the noise hazard problem is becoming more serious. In a platinum mine in South Africa, more than 80% of miners were exposed to noise exposure levels that were higher than 85 dB(A), with 64% of the miners having higher noise exposure than 91 dB(A) (5). Sixty-nine percent of workers in sand and gravel mines were exposed to noise above the exposure limits recommended by NIOSH (6). A survey of hard-rock miners in the western United States found that 96% of operators had daily noise doses of more than 90 dB(A) (7). Measured 102 dB(A) from underground mining in the mining industry in Zimbabwe (8). Another study of Tanzanian miners also suggested that high noise exposures were common among miners (9). In China, a study of three metal mine enterprises found that the average individual noise is above 89.7 dB(A), especially the noise of drilling workers above 102.5 dB(A) (10). According to another study on six metal mines, 56.3% of the area noise exceeds 85 dB(A) (11). Among the eight non-coal mining enterprises in Dalian (five limestones and silica mining, three granite mining), 55.56% of the area noise exceeds 85 dB(A) (12). However, currently reported mining noise levels are primarily the result of one or more mines, with some studies only reporting the noise doses from areas or equipment, lacking assessments of workers' noise exposure. Extensive data on miner noise levels in China have rarely been reported. To address this problem, the National Institute for Occupational Health and Poison Control conducted a series of noise surveillance and evaluation studies for the non-coal mining industry. This research effort was conducted at 82 non-coal mines in four provinces to determine miners' noise exposure levels. Four hundred twenty-three noise exposure measurements were obtained from mining workers.

## Materials and methods

### Study setting

This research is analytical in the form of an observational study. Due to China's uneven geographical distribution of mineral resources, this study adopted the typical sampling method and selected 82 non-coal mines in four provinces. Non-coal mines refer to mines other than coal mines, which mainly include metal and non-metal mines. This exposure study was conducted among workers in non-coal mines to evaluate their noise exposure, and a noise dosimeter was used to measure workers' noise exposure during their work day. The study started in January 2019 and ended in December 2020.

### Description of the production process

Mining is divided into surface mining and underground mining. The part close to the surface and buried shallowly adopts surface mining, and the deep part adopts underground mining. The production process is divided into six sections: excavation (drilling and blasting), ore drawing, transportation, winching, beneficiation (crushing, screening, and ball milling), and auxiliary (air pressure, pump, and maintenance). According to the production process, the noise mainly comes from the aerodynamic noise of pneumatic rock drilling tools, the mechanical noise generated by vibration, friction, and collision of various equipment during operation, and the electromagnetic noise caused by electrical equipment. The noise-related equipment includes air compressors, various pumps, fans, winches, blasting equipment, crushing equipment, rock drilling equipment, transportation equipment, rock loaders, and machine repair equipment. The workers were categorized according to job titles and descriptions (Table 1).

**Table 1:** Summary of job descriptions by job title.

**Job title**	**Job description**
Excavation worker	They are excavating roadways, including drilling and blasting.
Miner	Mining the ore from the face and transporting it to the pit (referring to underground mining) or steps (referring to surface mining)
Winch operator	Operate the winch to lift heavy objects
Belt operator	Operate the belt conveyor and ensure the regular operation of the ore transport belt
Crusher operator	Operate the crusher to break large chunks of ore into smaller pieces
Screening operator	Operate the screening machine to separate the mixed ore of different sizes into various particle size classes
Mill operator	Operate the mill to pulverize the ore
Pump operator	Operation and maintenance of various pump equipment
Air compressor operator	Operation and maintenance of air compressors and air supply
Transporter	Operate forklifts, transport vehicles
Control worker	Monitor and oversee work activities
Maintenance worker	Maintenance and repair of mining equipment

### Individual noise measurement

To ensure the validity and authenticity of the measurement, we selected workers who had been in their current jobs for more than 1 year as participants and determined before the measurement that workers could work an entire shift. The shift-long individual noise exposure was measured for each participant using a Casella dBadge2 individual noise dosimeter. The dosimeter microphone was placed at the midpoint of the participants' shoulders and worn throughout the work shift. The dosimeters were equipped with a single ½-inch microphone, the dynamic range of the dosimeters was 55.0–140.3 dB(A), and the exchange rate was 3 dB. Before the measurement, each dosimeter was calibrated using the Casella 120/2 Acoustic Calibrator. Each dosimeter was used to detect a complete work shift. Individual noise exposure measurements were performed for all operational jobs, with 1–3 participants were selected to detect three shifts for the jobs. The measurement recorded was the normalization of equivalent continuous A-weighted sound pressure level to a normal 8 h working day (L_EX,8h_) or a nominal 40 h working week (L_EX,40h_). The noise level exposed to 5 days per week was equivalent to L_EX,8h_, and the noise level exposed to non-5 days per week was equivalent to L_EX,40h_. The L_EX,8h_ and L_EX,40h_ were calculated by the formula in ISO 1999:2013:


$$\begin{array}{l} L_{EX,\;8\;h}=\;L_{Aeq,\;T_{e}}+10\mathrm{lg}\left(\frac{T_{e}}{T_{0}}\right)\;dB \end{array}$$

where T_e_ is the effective duration of the working day in hours; T_0_ is the reference duration (T_0_ = 8 h); and L_Aeq, Te_ is the L_Aeq_ for T_e_.


$$\begin{array}{l} L_{EX,\;40\;h}=\;10\mathrm{lg}\left(\frac{1}{5}\overset{n}{\underset{i=1}{\sum }}10^{0.1\left(L_{EX,\;8\;h}\right)\;i}\right)\;dB \end{array}$$

where n is the actual number of working days per week; L_EX,8h_ is the noise exposure level normalized to a nominal 8 h working day.

According to GBZ2.2-2007 (13), individual noise exposure should not exceed 85 dB(A). This level is defined as the permissible exposure level (PEL). These measurements include information about the mine region, scale, type (surface or underground), and mining content (metal or non-metal), as well as the job title or task description for each measurement. Moreover, we collected information on employers equipping HPD for their workers.

### Risk assessment of occupational noise-induced hearing loss

ISO 1999:2013(E) (14) is an international standard for risk assessment of occupational noise-induced hearing loss (NIHL), which can be applied to the calculation of the risk of sustaining hearing loss due to regular occupational noise exposure. In statistical terms, it presents the relationship between noise exposures and the “noise-induced permanent threshold shift” (NIPTS) in people of various ages.

The hearing threshold level associated with age and noise (HTLAN), H′, can be calculated by the formula in ISO 1999:2013:


$$\begin{array}{l} H^{{}^{\prime }}=H+N-\frac{H\times N}{120} \end{array}$$

where *H* is the hearing threshold level associated with age (HTLA), expressed in decibels; *N* is the actual or potential noise-induced permanent threshold shift (NIPTS), expressed in decibels.

ISO 1999:2013 permits two databases (databases A and B) to be used for the hearing threshold level associated with age (HTLA). Database A was derived from otologically normal persons. In this study, database A was selected to predict HTLA changes in 10, 50, and 90% of workers.

All mine workers in the study were male. We assumed the workers started working at the age of 20 and worked on a job for 40 years.

According to the Diagnosis of Occupational Noise Deafness (GBZ 49-2014) (15), the definitions, frequencies, and fences of high-frequency hearing loss and noise-induced deafness were determined. High-frequency hearing loss was defined as an average hearing threshold of bilateral high-frequency (3,000, 4,000, 6,000 Hz) ≥ 40 dB. The frequencies of 3,000, 4,000, and 6,000 Hz (1/3 of each) were selected, and 40 dB was set as the fence. Noise-induced deafness was defined as the optimal whisper frequency, and the weighted value of 4,000 Hz in high-frequency hearing loss ≥26 dB. The frequencies of 500, 1,000, 2,000, and 4,000 Hz (ratio 3:3:3:1) were selected, and 26 dB was set as the fence.

The ISO1999 formula was used to calculate the HTLAN of 10, 50, and 90% of the workers and NIPTS of 10, 50, and 90% of the workers. Based on the noise exposure level of the corresponding job, the changes in the hearing threshold level of workers of each position after 40 years of work were predicted. The risk of high-frequency hearing loss and noise-induced deafness were calculated for workers in different jobs at the same age (60 years old) and exposure years (40 years of working). The risk of NIHL was represented by the percentage of people whose NIPTS, HTLAN, and HTLA exceeded the selected fence.

### Statistics

The data of the noise dosimeter were checked for validity, and invalid data with battery failure or incorrect settings were eliminated. Statistical analysis was performed using SPSS (version 22.0, SPSS Inc., Chicago, IL, USA). The median (M), interquartile range (**P**_**25**_**, P**_**75**_), and percentage of measurements with levels above PEL were calculated to describe the distribution of the noise exposure level, and the chi-square test or Fisher's exact test was applied to analyze the difference in individual noise exposure levels in non-coal mines among mineral type, mining mode, and scale. The HPD equipment was also obtained from the survey of mining enterprises to analyze the differences in mineral type, mining mode, and scale. A significant difference was considered when *P* < 0.05.

### Ethical approval

Clearance was issued by the National Institute for Occupational Health and Poison Control, the Chinese Center for Disease Control and Prevention (NIOHP, China CDC). This study did not cause any physical or psychological harm or disturb the operators during the operation. Informed consent was obtained from all individual participants involved in the study. Information on HPD was obtained from communication with company occupational health managers and confirmed during this investigation.

## Results

Table 2 shows the results of the non-coal mine workers' noise data. Noise dosimeter measurements (L_EX,8h_/L_EX,40h_) were recorded for 423 workers in non-coal mines. The median individual noise exposure was 83.4 dB(A). The worker dose measurements indicated that 31.9% (135/423) of all measurements were above the PEL of 85 dB(A).

**Table 2:** Distribution of noise exposure among non-coal mining workers in four provinces of China.

**Group**	***N***	Individual noise exposure level L_EX,8h_/${\text{L}}_{\text{EX},40\text{h}}^{*}$[dB(A)] M (P_25_, P_75_)	**The proportion of individual noise exposure levels ≧85 dB(A) *N* (%)**	**χ^2^**	***P***
Total	423	83.4 (79.6, 86.4)	135 (31.9)		
Job				—	—
Excavation worker	67	89.1 (84.9, 96.6)	48 (71.6)		
Miner	134	82.7 (81.1, 84.6)	22 (16.4)		
Winch operator	16	80.8 (79.3, 82.4)	1 (6.3)		
Belt operator	23	82.8 (79.7, 86.6)	9 (39.1)		
Crusher operator	51	87.0 (85.0, 89.2)	38 (74.5)		
Screening operator	19	84.1 (83.4, 84.5)	2 (10.5)		
Mill operator	11	88.7 (88.3, 91.0)	10 (90.9)		
Pump operator	8	73.4 (72.2, 76.0)	0 (0)		
Air compressor operator	9	77.8 (77.1, 78.1)	0 (0)		
Transporter	63	80.8 (78.8, 83.0)	4 (6.3)		
Control worker	14	76.1 (72.5, 77.9)	0 (0)		
Maintenance worker	8	76.8 (75.7, 84.7)	1 (12.5)		
Mineral type				3.340	0.068
Non-metal	231	83.5 (80.9, 85.6)	65 (28.1)		
Metal	192	83.0 (78.2, 87.9)	70 (36.5)		
Mining mode				10.964	0.001
Underground	144	84.1 (79.6, 90.6)	61 (42.4)		
surface	279	83.3 (79.6, 85.4)	74 (26.5)		
**Scale** **(****16****)**				6.103	0.107
Large	25	86.2 (76.6, 92.2)	13 (52.0)		
Medium	100	83.0 (80.6, 87.2)	35 (35.0)		
Small	152	81.8 (78.1, 86.5)	44 (28.9)		
Micro	146	83.8 (82.0, 85.7)	43 (29.5)		

^*^L_Ex,8h_: Normalization of equivalent continuous A-weighted sound pressure level to a nominal 8 h working day. L_Ex,40h_: Normalization of equivalent continuous A-weighted sound pressure level to a nominal 40 h working week.

This table shows the distribution of noise exposure among non-coal miners. This illustrates that the noise dose range within different jobs varies considerably. The individual noise exposure levels of the pump operators, air compressor operators, control workers, and maintenance workers involved in the test were lower than 80 dB(A). Mines, winch operators, belt operators, screening operators, and transporters were exposed to noise between 80 and 85 dB(A). Excavation workers, crusher operators, and mill operators were exposed to high doses of noise, with 89.1 dB(A) for excavation workers, 88.7 dB(A) for mill operators, and 87.0 dB(A) for crusher operators.

Mining mode (underground or surface) had a significant effect on the noise exposure of workers (*P* < 0.001). The noise exposure was significantly higher in underground mines than in surface mines.

According to ISO 1999:2013, there was a risk of NIHL for workers exposed to 80 dB(A) sound. This NIHL risk was assessed in Table 3 for the workers of this study.

**Table 3:** The estimated risk of hearing loss during 40 years of working in workers who are exposed to noise above 80 dB(A) based on ISO 1999:2013.

**Group**	**L_EX,8h_ [dB(A)]**	**Estimated NIHL risk of high-frequency hearing loss (%)**	**Estimated NIHL risk of noise-induced deafness (%)**
**Job (exposed to noise over 80 dBA)**			
Excavation worker	89.1	14.3	4.5
Miner	82.7	2.9	0.6
Winch operator	80.8	1.1	0.2
Belt operator	82.8	3.1	0.7
Crusher operator	87.0	9.7	2.7
Screening operator	84.1	4.7	1.1
Mill operator	88.7	13.4	4.1
Transporter	80.8	1.1	0.2
**Mineral type**			
Non-metal	83.5	3.9	0.9
Metal	83.0	3.3	0.7
**Mining mode**			
Underground	84.1	4.7	1.1
Surface	83.3	3.7	0.8
**Scale**			
Large	86.2	8.2	2.2
Medium	83.0	3.3	0.7
Small	81.8	2.0	0.4
Micro	83.8	4.3	1.0

Enterprises must provide hearing protection for noise-exposed workers, especially for employees exposed to >85 dB(A). Based on Table 4, only 46.3% (38/82) of mining enterprises had equipped HPD for their workers. More than half of mining employers had never been equipped with HPD for their workers (53.7%). The proportion of non-metallic mines (74.5%), surface mining (69.4%), and small or micro-mining enterprises (59.5%) not equipped with HPD is relatively high.

**Table 4:** The equipment of HPD in non-coal mining enterprises in four provinces of China.

**Group**	**Number of mines**	**HPDs**		**χ^2^/Fisher**	***P*-value**
		**Equip**	**Un-equip**		
Total	82	38 (46.3)	44 (53.7)		
Mineral type				29.306	< 0.001
Non-metal	55	14 (25.5)	41 (74.5)		
Metal	27	24 (88.9)	3 (11.1)		
Mining mode				25.186	< 0.001
Underground	20	19 (95.0)	1 (5.0)		
Surface	62	19 (30.6)	43 (69.4)		
Scale (16)				10.265^*^	0.001
Large and medium	8	8 (100)	0		
Small and micro	74	30 (40.5)	44 (59.5)		

^*^Fisher's exact test.

## Discussion

This study found high levels of hazardous noise exposure in the sampled non-coal mines from four provinces in China. Workers were exposed to a median noise level of 83.4 dB(A), with 31.9% of individual noise measurements exceeding the PEL of 85 dB(A).

In this survey, excavation workers, mill operators, and crusher operators were exposed to high noise levels; 71.6–90.9% exceeded 85 dB(A). Among them, excavation workers were the job with the highest noise exposure in non-coal mines. Armah et al. (17) stated that the maximum average level of cubic operators (drill service holes and production of slots) was 103.9 dB, and Lutz et al. (18) found that the noise exposure for jumbo drill operation was 103.0 ± 0.8 dB(A). In this study, the highest noise level of excavation workers in the underground mine was 103.2 dB, and the results were close to the studies above. These workers are located close to large, noisy equipment for long periods and are chronically affected by noise levels that have the potential to cause NIHL.

According to ISO 1999:2013, it is predicted that excavation workers, mill operators, and crusher operators in this survey had the highest NIHL risk over a 40-year working life, with a 60-year-old male exposed to noise at a level of 87.0 dB(A) for 40 years having a 9.7% risk of high-frequency hearing loss and a 2.7% risk of noise-induced deafness. At a level of 88.7 dB(A), the risk of high-frequency hearing loss was 13.4%, and the risk of noise-induced deafness was 4.1%. At a level of 89.1 dB(A), there was a 14.3% risk of high-frequency hearing loss and a 4.5% risk of noise-induced deafness. Hearing loss requires long-term exposure to hazardous noise levels before a significant decline in hearing levels can be noticed. The prevalence of NIHL increased with higher noise levels and higher duration of exposure (19). In the case of the same gender, age, and working years, the predicted hearing loss depended entirely on the noise exposure intensity of workers, which increased with the increase in individual noise exposure. The prediction results of the ISO 1999:2013 model were consistent with the development law of hearing loss. These jobs (excavation worker, mill operator, and crusher operator) were likely to have a high incidence of occupational hearing loss, which was consistent with the high noise exposure positions identified in other studies (20, 21).

Previous studies have shown that the prevalence of hearing loss among workers in the mining industry in the United States was 27.3% (22). The prevalence rates of high-frequency hearing loss and noise-induced deafness hearing loss among blasting, excavation and mining workers in a mining enterprise in China were 73.52 and 13.11%, respectively (23). Among three non-ferrous metal mines in Gansu Province, 41.84% of workers suffered from hearing loss (24). Zhang et al. (25) investigated 25 outdoor quarries and found that 54.1% of workers suffered from hearing loss. The prevalence of NIHL in the above studies was higher than the predicted result by ISO 1999:2013. Research has shown that the prediction of NIHL by ISO 1999:2013 may be underestimated (26–28). There were many reasons for this underestimation. First, ISO 1999:2013(E) used a single noise-equivalent sound level as an evaluation index, which could not adequately reflect the exposure level of complex noise (27, 29, 30). Second, non-occupational noise exposure was ignored in the ISO 1999:2013 model. Noise-induced hearing loss is not only a part of occupational noise exposure but also important in non-occupational exposure. The use of earphones has been a major concern in studies on non-occupational noise exposure. Listening to music with headphones for a long time and loud volume will lead to hearing loss (31–33). In addition, co-exposure to noise and chemicals resulted in greater hearing loss than noise exposure alone (34, 35). However, since hearing loss is a process of gradual development, the application of ISO 1999:2013 in the risk assessment of high-frequency hearing loss can be used as an early warning method of hearing loss to find the potential risk of hearing loss in the population.

The noise exposure levels of miners, winch operators, belt operators, screening operators, and transporters ranged from 80 to 85 dB. ISO 1999: 2013 predicted that the risk of high-frequency hearing loss in these male workers over a 40-year working life ranged from 1.1 to 4.7%, and the risk of noise-induced deafness ranged from 0.2 to 1.1%. The investigation revealed that the transporters operate in the cab, the winch operators also work in the dedicated operating room, and their environment is relatively closed to reduce noise exposure. A study of construction equipment operators confirmed that operators were exposed to less noise with the cab's proper design and the cab's insulation (36).

The studied pump operators, air compressor operators, and control workers were exposed to individual noise levels below 80 dB(A). The operating mode of these workers is inspection, with ~2 h of inspection per shift, and they spend more time in the quiet duty or control room.

Workers in underground mines are exposed to more noise than in surface mines (*P* < 0.001). Compared to surface mines, underground mines operate in relatively confined spaces with equipment closer to operators, resulting in higher noise exposure for miners. This result was supported by the Mine Safety and Health Administration (MHSA), which confirmed that underground metal mining has the highest noise exposure of all mine types (37). The data from MSHA indicated that the mean exposure for an 8-h time-weighted average was 81.9 dB(A) in metal mines and 82.1 dB(A) in non-metal mines (37). The results of this study showed that the noise exposure of metal mines was 83.0 dB(A) and that of non-metal mines was 83.5 dB(A), which is slightly higher than the results of MSHA, but there is no significant difference in noise exposure between metal mines and non-metal mines (*P* > 0.05).

Larger mines may have tended to use more powerful mining equipment, but the differences in noise exposure doses for workers in mines of different sizes were not significant (*P* > 0.05).

HPDs such as earplugs and earmuffs are low-cost and straightforward noise mitigation devices. A total of 53.7% of mining enterprises did not equip personal hearing protective devices for workers. Although there is no significant difference in noise exposure between small and micro-sized mining enterprises and large and medium-sized enterprises, the HPD equipment rate of small and micro-sized enterprises is only 40.5%, which is much lower than that of large and medium-sized enterprises.

The rate of HPD equipment in underground mines was significantly higher than that in surface mines (*P* < 0.001), and 95% of underground mining enterprises provided HPD for workers. On the one hand, it shows that the occupational health management of underground mines is better than that of surface mines, and on the other hand, it indirectly shows that the noise hazards of underground mines cannot be ignored.

From the perspective of mineral type, the HPD equipment rate of non-metallic mines is much lower than that of metal mines, only 25.5%. Landen et al. (6) found that hearing protection usage was low among sand and gravel miners, with 48% of workers reporting that they never used hearing protection. Sun and Azman (38) also found that stone, sand, and gravel mines at surface operations exposed a more significant number of miners to excessive risk, and management commitment to hearing loss prevention was low. The non-metallic mines in this survey were surface mines, 98.2% of which are small and micro enterprises. The mining mode and scale of the mine may be the main reasons for the low HPD equipment rate of non-metallic mines. These results suggest that noise control and management in small mines and micro mines should be improved to reduce overexposure before these workers develop occupational hearing loss.

The study did not investigate workers' actual use of HPD, but the reality is not optimistic. Studies have shown (39) that < 50% of workers use hearing protectors in a large gold mine in South Africa. The use of HPD can effectively reduce the noise exposure dose of workers, but how to improve the use of HPD has been difficult to solve. Occupational noise-related policies can positively impact hearing protection and increase the use of HPD (40, 41).

Controlling noise exposure is the fundamental method of protecting workers from high noise exposure risk. According to the NIOSH information, the hierarchy of controls was recommended to determine feasible and effective controls to implement, including elimination, substitution, engineering controls, administrative controls, and personal protective equipment (PPE) (42). To protect the hearing health of non-coal workers, improving the production processes and implementing automation could be considered to reduce the intensity of noise generated from equipment. For equipment that generates noise at a high intensity, engineering control measures should be taken, such as muffling or adding sound insulation, reducing the impact and friction of machinery, or controlling the length of stay in these high-noise environments. PPE can provide worker protection when other levels of control combined do not adequately eliminate noise hazards.

The Chinese government attaches great importance to preventing and controlling workers' occupational disease hazards and has formulated regulations and norms related to occupational health. Occupational health-related laws and regulations require employers to provide hearing protection and appropriate training for noise-exposed workers. The focus of the next step in noise protection should be to strengthen the management and supervision of hearing protection in small and micro-sized mining enterprises. In addition to providing workers with appropriate HPD as required by laws and regulations, employers need to monitor the proper use of HPD closely.

The findings in this report are subject to at least two limitations. Subjects were from selected areas, and the summary of the findings may be limited. Nevertheless, we obtained 423 individual dosimeter measurements. To our knowledge, no previous studies conducted in non-coal mines have been able to obtain a similar number of dosimeter measurements. Second, during the surveillance, we did not acquire information on workers' occupational health.

## Conclusion

Noise Exposure continues to be a problem in non-coal mines. In this study, non-coal mining workers at different scales, mineral types, and modes were examined by placing a noise dosimeter on the shoulders of mining workers during an entire work shift. More than 31.9% of the non-coal mining workers were exposed to noise higher than 85 dB(A), which can seriously affect human health. The HPD equipment rate of small and micro-sized enterprises is only 40.5%, indicating that small and micro-sized enterprises have an insufficient investment in noise control. It is necessary to focus on strengthening the management and supervision of hearing protection in small and micro-mining enterprises.

## Data availability statement

The original contributions presented in the study are included in the article/supplementary material, further inquiries can be directed to the corresponding author.

## Ethics statement

The studies involving human participants were reviewed and approved by the National Institute for Occupational Health and Poison Control, the Chinese Center for Disease Control and Prevention. The patients/participants provided their written informed consent to participate in this study.

## Author contributions

XW: investigation, formal analysis, and writing-original draft. NK, YD, and KN: methodology and investigation. KL: investigation and data processing. HB, FH, and YC: formal analysis. MY: conceptualization, funding acquisition, writing—review and editing, and supervision. All authors contributed to the article and approved the submitted version.
